# Correction: LFA-1 mechanical dose controls DNAM-1 signalling and perforin polarization in NK cells

**DOI:** 10.1039/d6ra90070d

**Published:** 2026-07-17

**Authors:** Seong Ho Kim, Micah Yang, Isaac T. S. Li

**Affiliations:** a Department of Chemistry, The University of British Columbia Kelowna BC V1V 1V7 Canada seonghok@kangwon.ac.kr isaac.li@ubc.ca; b Department of Chemistry and Advanced Materials, Kangwon National University Gangneung 25457 Republic of Korea

## Abstract

Correction for ‘LFA-1 mechanical dose controls DNAM-1 signalling and perforin polarization in NK cells’ by Seong Ho Kim *et al.*, *RSC Adv.*, 2026, https://doi.org/10.1039/d6ra02527g.

The authors regret that due to an unexpected change to funding information during the publication of this paper, the grant details provided in the acknowledgments are no longer accurate.

The correct acknowledgments are as follows:

The research is supported by the Natural Sciences and Engineering Research Council of Canada (NSERC) Discovery Grant (RGPIN-2024-04352), Canada Research Chairs (CRC-2020-00143), Michael Smith Health Research BC (SCH-2020-0559, RT-2023-3286), Canada Foundation for Innovation (35492, 40086, 41104), research funds for newly appointed professors of Kangwon National University in 2025, and the ANCHOR program through the Gangwon ANCHOR Center, funded by the Ministry of Education(MOE) and the Gangwon State (G.S.), Republic of Korea (2026-ANCHOR-10-004).

The Royal Society of Chemistry apologises for these errors and any consequent inconvenience to authors and readers.

